# Update on the Neuroimaging and Electroencephalographic Biomarkers of Epileptogenesis: A Literature Review

**DOI:** 10.3389/fneur.2021.738658

**Published:** 2021-08-27

**Authors:** Guihua Chen, Zheyu Zhang, Meiping Wang, Yu Geng, Bo Jin, Thandar Aung

**Affiliations:** ^1^Department of Neurology, Zhejiang Provincial People's Hospital, People's Hospital of Hangzhou Medical College, Hangzhou, China; ^2^The Second Clinical Medical College, Zhejiang Chinese Medical University, Hangzhou, China; ^3^Epilepsy Center, University of Pittsburgh, Pittsburgh, PA, United States

**Keywords:** epilepsy, epileptogenesis, biomarker, neuroimage, EEG

## Abstract

Epilepsy is one of the most common debilitating neurological disorders that lead to severe socio-cognitive dysfunction. While there are currently more than 30 antiseizure medications available for the treatment and prevention of seizures, none address the prevention of epileptogenesis that leading to the development of epilepsy following a potential brain insult. Hence, there is a growing need for the identification of accurate biomarkers of epileptogenesis that enable the prediction of epilepsy following a known brain insult. Although recent studies using various neuroimages and electroencephalography have found promising biomarkers of epileptogenesis, their utility needs to be further validated in larger clinical trials. In this literature review, we searched the Medline, Pubmed, and Embase databases using the following search algorithm: “epileptogenesis” and “biomarker” and “EEG” or “electroencephalography” or “neuroimaging” limited to publications in English. We presented a comprehensive overview of recent innovations in the role of neuroimaging and EEG in identifying reliable biomarkers of epileptogenesis.

## Introduction

Epilepsy is one of the most common neurological disorders affecting around 70 million people worldwide. Approximately 2.4 million new cases of epilepsy are diagnosed annually (1). Subsequently, epilepsy is getting increased public health attention as patients with epilepsy have a noticeable reduction in quality of life and employment prospects (2). Although antiseizure medication (ASMs) are considered the first-line treatment of epilepsy, it is still widely recognized that nearly one-third of epileptic patients have drug-resistant epilepsy in which seizures are unable to be controlled with at least two appropriately ASMs (3). One potential reason is that current accessible ASMs merely prevent one from having further spontaneous seizures but do not directly affect or alter the underlying cause contributing to epileptogenesis (4, 5). Through plentiful scientific research on the pathophysiology of epilepsy over the past several decades, there has been an increasing understanding of the pathophysiology of epileptogenesis.

Epileptogenesis refers to the process by which normal brain tissue becomes capable of generating spontaneous recurrent seizures and its progression (6). With the advances in technology, potential promising biomarkers are now available to predict the development of epileptogenesis after the epileptogenic insult. Furthermore, there are accessible therapeutic biomarkers that predict the treatment prognosis through the identification and exact localization of the epileptogenic lesion and associated network, as well as the severity and progression of epileptogenesis (7, 8). The ideal biomarkers should not just have validity, reliability, and reproducibility; they should also be non-invasive and cost-effective (9). Among all the available biomarkers related to epileptogenesis, neuroimaging and electroencephalogram (EEG) biomarkers are by far the most appealing biomarkers as they are non-invasive and routinely performed as part of epileptic patients' workup protocol.

In this review article, we searched the Medline, Pubmed, and Embase databases using the following search algorithm: “epileptogenesis” and “biomarker” and “EEG” or “electroencephalography” or “neuroimaging” limited to publications in English. The last date of the search was May 31, 2021. We screened the titles, abstracts, and references of all search results to identify potentially relevant studies. We aimed to provide a comprehensive literature review of recent innovations in the role of neuroimaging and EEG as biomarkers of the development of epileptogenesis after the epileptogenic insult.

## Neuroimaging Biomarkers

Several neuroimaging modalities, including structural magnetic resonance imaging (MRI), functional MRI, magnetic resonance spectroscopy (MRS), and positron emission tomography (PET), have already been applied to investigate biomarkers of epileptogenesis and have significantly contributed to our understanding of the pathophysiological mechanisms that underlie the development of epilepsy (Table 1).

**Table 1:** Overview of neuroimaging biomarkers of epileptogenesis.

**Imaging modality**	**Animal model**	**Human epilepsy**	**References**
*Structural MRI*
Surface morphology	LFPI model	None	(10)
D_av_	LFPI model	None	(11)
Cortical thinness	None	PTE	(12)
PVSs	None	PTE	(13, 14)
T2 relaxation time	FSE	None	(15, 16)
DWI	None	Children with FSE	(17)
DTI	LFPI model	None	(18)
	Pilocarpine -induced SE mode	None	(18)
	MSO infused model	–	(19)
*Functional MRI*			(20)
Global network	KA-induced SE model	None	(21–23)
Local network	KA-induced SE model	None	(21)
*MRS*
NAA	Pilocarpine -induced SE model	None	(24, 25)
GABA-A	Pilocarpine -induced SE mode	None	(26)
Myo-inositol	Pilocarpine -induced SE mode	None	(25, 27)
Antioxidant glutathione	Pilocarpine -induced SE mode	None	(25)
*PET*
18F-FDG	LFPI model	None	(10)
	KA-induced SE model	None	(23–32)
	Pilocarpine-induced SE model	None	(30)
GABA-A	KA-induced SE model	None	(33–35)
TSPO	KA-induced SE model	None	(36, 37)

*LPFI, lateral fluid percussion injury; PTE, posttraumatic epilepsy; PVSs, paravscular spaces; DWI, diffusion-weighted imaging; FSE, febrile status epilepticus; DTI, diffusion tensor imaging; KA, kainic acid; SE, status epilepticus; MSO, methionine sulfoximine; NAA, N -acetyl aspartate; TSPO, 18-kDa translocator protein*.

### Structural MRI

MRI is an ideal tool for biomarker studies due to its accessibility and translatability to routine clinic settings. In the lateral fluid percussion injury (LFPI) rat model of traumatic brain injury (TBI), the abnormalities in the surface morphology of the ipsilateral hippocampus at 1-week post-LFPI can predict the occurrence of epilepsy 6 months after TBI (10). Besides, assessing individual MRI parameters in the peri-lesional cortex or the thalamus at 9 days after TBI can also provide high sensitivity and specificity for predicting increased seizure susceptibility at 12 months (11). In the follow-up LFPI study, the presence of diffusion abnormality analyzed by using D_av_ in the perilesional cortex and thalamus at 2 months after the TBI is found to have the highest predictive value for the development of seizure susceptibility at 12 months post-TBI. Similar changes in the MRI structures have been validated at the human level. In the early acute post-TBI phase (within 90 days post-injury), there is evidence showing a positive correlation between hippocampal/temporal structural abnormalities and the onset of seizure activity (11). In addition to the injury severity, the left temporal pole and left frontal cortical thinness are found to be significantly predictive factors for developing seizures after TBI (12). In addition to the cortical and subcortical structures, studies have shown that the distribution and quantification of paravscular spaces (PVSs) can be used as a potential biomarker for the development of epileptogenesis in posttraumatic epilepsy (PTE). Post-TBI epileptic patients are found to have significantly smaller PVSs and asymmetric distribution of PVSs in the suspected epileptogenic hemisphere (13, 14).

Mesial temporal lobe epilepsy (TLE) is reported as a common sequelae of the febrile status epilepticus (FSE) (38). Recently, in the rat model of FSE, reduced T2 relaxation time in the amygdala within 2 h of FSE is observed in the high resolution 11.7T MRI. This finding is shown to have a strong prediction of the later occurrence of TLE following the FSE. It is hypothesized that T2 changes are related to the increased oxygen utilization after FSE termination, which correlates with the activation of the intracellular inflammatory cascades that had been previously implicated in epileptogenesis (15). The result is again validated in the lower resolution 3T MRI (16). These results suggest that the quantitative T2 MRI can be used as a reliable neuroimaging biomarker following FSE for brain injury and structural alterations at the onset of epileptogenesis. Further studies are warranted to validate the reduction of the T2 relaxation time in the amygdala in the development of epileptogenesis in humans before they are ready for the clinical setting. Overall the translational nature of the MRI results has also greatly contributed to clinical advancements as the reported neuro-imaging protocols can be applied safely to epileptic patients.

Diffusion-weighted imaging (DWI) represents the diffusion of water molecules and is particularly useful for detecting acute changes in the brain tissue following status epilepticus (SE) (17). Yokoi and colleagues analyzed the acute DWI data of 22 children with FSE over the period of 9–13 years following FSE and reported that focal epilepsy was significantly more frequent in patients with hippocampal DWI hyperintensity than those without DWI changes (39). Further studies, including a larger cohort, are required to investigate the relationship between DWI changes and subsequent epilepsy in patients with FSE.

Diffusion tensor imaging (DTI) detects the restriction of water diffusion caused by the microstructural organization of tissues. Advancement in post-processing technique allows DTI to detect subtle white matter changes in the early development of epileptogenesis in a structural network-based approach (40). Sierra and colleagues studied and compared the fractional anisotropy (FA) and axial, radial, and mean diffusivities among three subgroups of rats: SE, TBI, and normal controls, at 6–12 months post-injury using 9.4T MRI. FA in the hippocampi was significantly increased in the SE/TBI group compared to the normal control group. Regarding the diffusivities, there was an increase in the *D*_||_ (associated with axonal damage) after the SE, whereas a decrease in *D*_⊥_(associated with demyelination) was noted after the TBI in the subfield-specific hippocampi. Thus, the data suggest that the DTI method identifies not only subtle hippocampal changes and progression after epileptogenic brain injuries but also different brain insults based on the different diffusivities (18). Several recent longitudinal studies of the mesial TLE rate models demonstrated that the epileptogenic rats have significant changes in DTI-measured FA in the early stages. In addition, the FA changes in both gray and white matter progress over time as the animals transitioned from early to late epileptogenesis (19). Although the results suggest that DTI changes can be used as biomarkers of epileptogenesis, no prospective studies of epileptogenesis with DTI have been implemented in humans. Thus, further studies are warranted to implement the finding in the clinical setting.

### Functional MRI

Functional MRI detects hemodynamic changes in different parts of the brain by means of blood oxygen level-dependent (BOLD) sequences, an indirect non-invasive measure of neuronal activity (41). Contrary to measuring the structural connectivity with DTI, functional MRI measures functional connectivity between various brain regions. Using the animal intrahippocampal kainic acid (KA) model of mesial TLE, Li et al. compared the fMRI of animals with mesial TLE and animals without epilepsy at 1 week after SE. For global network features, animals with epilepsy showed an overall increase in functional connectivity strength compared to animals without epilepsy. For local network features, animals without epilepsy showed decreased hubness in the hippocampus, whereas animals with epilepsy showed a complete loss of hippocampus hubs with appearance of new hubs in the prefrontal cortex (21). Instead of the hypersynchrony brain network pattern, Christiaen et al. reported a decreased functional connectivity between 1 and 3 weeks post SE after comparing 20 intraperitoneal KA animals and seven healthy control animals (22). The difference might be the result of the different routes of administration of ketamine as intraperitoneal injection resulted in more widespread brain lesions than intrahippocampal injection (21). Furthermore, Bertoglio et al. demonstrated diverging changes in network connectivity in relation to the seizure onset in the KA-induced models of SE. Animals with regular seizure onset (<17 days post-SE) showed a significant hypersynchrony of network connectivity at 4 weeks post-SE, while animals with delayed disease onset (≥17 days post-SE) remained hyposynchronous (23). Although there is a discrepancy across different studies on functional connectivity after TBI or TLE, the current literature suggests that there may be a reorganization of the functional network in early period of epileptogenesis, which may be used as an imaging biomarker in the near future.

### MRS

MRS can provide indirect information, such as neuronal health, gliosis, energy metabolism, by analyzing different metabolites in the brain tissue. Several studies have analyzed the changes of N -acetyl aspartate (NAA) and gamma-aminobutyric acid (GABA) in the pilocarpine-induced SE model, a reduced NAA and GABA can be detected in the hippocampus from baseline to the period of epileptic seizures (24–26). However, a decrease in GABA and NAA levels has also been found in patients after TBI without correlation with epileptic seizures (28). A progressive increase in myo-inositol and antioxidant glutathione before the onset of seizures has been found in pilocarpine-induced SE (25, 27). Furthermore, the level of antioxidant glutathione has shown to be negatively correlated with the frequency of spontaneous seizures (25). Although MRS analyses in seizure-prone brain areas following potential epileptogenic injuries may represent clinically meaningful biomarkers for the early identification of individuals at high risk for developing epilepsy, further human studies are warranted to validate the finding for the clinical setting.

### PET

Alteration in the brain metabolic activity has been reported in the early development of the epileptogenesis after the initial epileptic insults. Nuclear imaging modalities such as PET are optimally used to assess functional brain metabolic activity using various radiotracers. Hence, PET has been used in several studies to assess potential mechanisms of epileptogenesis.

In the LFPI model, epileptic rats' ipsilateral hippocampi are reported to have subtle thickening on the surface analysis and 18F-FDG PET hypometabolism at 1 week, 1 month, and 3 months post-injury compared to the non-epileptic group. In addition, all the TBI rats have reported cortical and hippocampal hypometabolism, but the non-epileptic group has a partial recovery of the FDG uptake at 3 months post-injury (10). Thus, an initial reduction in glucose uptake is perhaps the result of both injury itself and early epileptogenesis, but in epileptic rats, there would be no recovery of the initial hypometabolism (29).

Multiple studies using KA and pilocarpine-induced models of SE have shown an initial increase in the glucose uptake during the acute seizures followed by the reduced metabolism at around day 3 of post-SE (24, 30). Furthermore, in the KA models of SE, glucose hypometabolism during early epilepsy correlates with the duration of the latent phase and frequency of spontaneous seizures in the spontaneous recurrent seizure (SRS) model of epilepsy (31, 32).

Several studies used different PET radioligands other than 18F-FDG to investigate the relationship between the density of GABA-A receptors and epileptogenesis in animal models of epilepsy. It is widely observed that GABA-A receptor density is decreased not only in hippocampi but also in several cortical regions in the KA models of SE (33, 34). One study using the focal cortical dysplasia model suggests that the decrease in the GABA-A receptor density may characterize a latent phase of epileptogenesis (35). Further studies are warranted to get more validation, but the promising results suggest that glucose hypometabolism and reduced GABA-A receptors might be the important hallmarks of early epileptogenesis.

Neuroinflammation is another pathological hallmark in one of the major pathophysiologies for epileptogenesis (42). The investigation of inflammation can be performed using a PET scan with radioligands that bind to18-kDa translocator protein (TSPO). TSPO is reported to be highly expressed on the mitochondrial membrane of activated microglia and reactive astrocytes. In the SRS model of epilepsy, TSPO levels at 14 days post-SE are predictive of SRS frequency at the onset of epilepsy (36). Subsequently, the same researcher group reported that TSPO upregulation at 14 days post-SE was associated with epileptogenesis, while TSPO overexpression at 14 days post-SE was associated with seizure frequency (37). Although TSPO-PET results are promising, clinical PET data on this topic is very limited due to the cost and availability of specific radioligands.

### EEG

EEG, either non-invasive scalp recording or invasive microelectrode recording, is one of the most utilized modalities in the clinical setting and can monitor brain activity with high temporal resolution and relatively high spatial resolution. Several studies have demonstrated specific changes in the EEG as the potential biomarkers for the early development of epileptogenesis (Table 2).

**Table 2:** Overview of EEG biomarkers of epileptogenesis.

**EEG biomarkers**	**Animal model**	**Human epilepsy**	**References**
HFO	KA-induced SE model	None	(43)
rHFOSs	LFPI model	None	(20, 44)
Scalp ripples	None	Children after a first unprovoked seizure	(45)
Sleep spindles	LPFI model	None	(46)
Theta band	Multiple rat and mouse models	None	(47)
Epileptiform activity	None	PTE and TSC	(48–51)
Background asymmetry	None	Epilepsy after stroke	(52)

*HFO, High-frequency oscillations; rHFOSs, repetitive HFO and spikes; KA, kainic acid; SE, status epilepticus; LPFI, lateral fluid percussion injury; PTE, posttraumatic epilepsy; TSC, tuberous sclerosis complex*.

### High-Frequency Oscillations (HFOs)

HFOs, i.e., ripples (80–250 Hz) and fast ripples (250–600 Hz), have been studied and shown to be promising biomarkers for epileptogenesis markers over the last decade. In the KA-induced SE model, Bragin et al. reported the appearance of HFOs in the ipsilateral hippocampi dentate nuclei in rats that later developed epilepsy. The author also described that the appearance of the HFO timing was found to be associated with the delay in the onset of the first seizure (43). The same group reported different types of HFOs named repetitive HFO and spikes (rHFOSs), in which rhythmic spikes at the frequency of 10–16 Hz with superimposed pathological HFOs (80–300Hz). The appearance of rHFOSs in the injured cortex and around the adjacent injured cortex within 2 weeks from the initial insult in the LFPI model rats was reported to more likely develop spontaneous seizures later in life (20, 44). Although HFOs are typically detected with invasive intracranial EEG, the advancements in scalp EEG monitoring equipment enable one to record HFOs on scalp EEG in human studies (53). In a cohort of children after a first unprovoked seizure, the presence of scalp ripples can predict the development of epilepsy (45). Further studies, including patients with TBI and febrile seizures, can enhance our knowledge of the role of scalp HFOs as biomarkers of epileptogenesis.

### Sleep Spindles and Theta Activity

Changes in the duration and frequency of the sleep spindles, one of the non-rapid eye movement stage II EEG features, have also been reported as a possible biomarker for epileptogenesis. In the LPFI model, Andrade et al. showed that shortening of the sleep spindles' duration and reducing of their frequency during slow-wave to rapid eye movement sleep transition can predict the development of epilepsy. Furthermore, receiver operating characteristics (ROC) analysis showed that spindle duration of <2.13 s (86% sensitivity, 80% specificity) and frequency of spindle <9.19 Hz (64% sensitivity, 60% specificity) could be used as biomarkers in differentiating rats with seizures from those without (46). These findings suggest that sleep spindles changes may be the indicators of widespread functional disturbance in the thalamocortical circuits following initial brain insult and could be used as a potential early biomarker for epileptogenesis.

Milkovsky et al. investigated the role of changes in the hippocampal dynamic in five animal models of epileptogenesis using the intrahippocampal recording. Among five frequency bands, changes in the dynamic of the theta band on days 2–4 post brain injury showed more than 90% sensitivity and specificity in predicting the animal which would develop epilepsy (47). Overall this finding is intriguing, but given the invasiveness nature of intracranial electrodes, further studies using non-invasive source modeling may help further validating the finding as early epileptogenesis in the human.

### Epileptiform Abnormalities and Background Asymmetry

The relation between epileptogenesis and scalp EEG findings such as interictal epileptiform discharges, lateralized periodic discharges, background EEG asymmetry, and electrographic seizures has been reported in humans. Although earlier studies in the 20th century do not give much yield in predicting the occurrence of epilepsy in patients with TBI (54), a recent retrospective study reported that during the acute traumatic phase, the presence of EEG interictal and ictal epileptiform abnormalities, such as sporadic interictal epileptiform discharges, lateralized or generalized periodic discharges, and seizures, are correlated with the development of the PTE at 1 year follow up (48). Punia et al. reported a similar result in adult patients that electrographic seizures or lateralized periodic discharges were related with the development of epilepsy after TBI (49). Two prospective multicenter studies investigated the role of scalp EEG in the infants with tuberous sclerosis complex. Among several EEG abnormalities, the author reported that the epileptiform discharges, not the hypsarrhythmia, showed the high positive predictive value and low negative predictive value in the development of epilepsy (50, 51). Furthermore, in addition to the interictal epileptiform activities, the presence of background asymmetry plays a role in the early development of epilepsy after stroke (52).

## Conclusions and Future Perspectives

Here we aimed to provide a comprehensive review of recent innovations in the role of neuroimaging and EEG as biomarkers of epileptogenesis after the epileptogenic insult. Identifying biomarkers of epileptogenesis would greatly facilitate not only diagnosis and treatment but also the early prevention of epilepsy in individuals at risk. Although several studies have identified potentially promising biomarkers for early epileptogenesis, such as changes in the amygdala T2 relaxation time, PVSs, TSPO-PET, global and local network connectivity reorganization, and HFOs, numerous challenges remain to implement the potential neuroimaging biomarkers to the bedside clinical setting. Firstly, the resolution capacity of human neuroimage is significantly lower than animal neuroimage. Thus, the same biomarker which is reported from animal studies may not be directly replicated in human studies. Secondly, each potential biomarker has both disadvantages and advantages, and it is unrealistic to expect that a single biomarker will epitomize the various types of epileptogenesis. Therefore, a combination of EEG and neuroimaging biomarkers might enhance the predictive power of epileptogenicity. Thirdly, the majority of published data are at the animal stage (summarized in Tables 1, 2). Before all these biomarkers can be utilized in the clinical setting, multicenter studies with standardized acquisition parameters and analysis procedures are needed to validate the robustness of biomarkers.

Currently, ongoing multicenter research studies are aiming to find biomarkers and treatments to prevent epileptogenesis. The European Union 7th Framework–funded project Targets and Biomarkers for Antiepileptogenesis (EPITARGET) is a consortium of 18 partners in nine European countries. In addition, the Epilepsy Bioinformatics Study for Antiepileptogenic Therapy (EpiBios4Rx), National Institute of Neurological Disorders and Stroke funded Centers without Walls study, is a collaborative multicenter international study conducted in the United States, Europe, and Australia. To data, biomarkers for epileptogenesis are still in the initial phase of the process. However, there will be more conclusive innovative EEG and neuroimaging early epileptogenic biomarkers and treatments from the multicenter trials to combat epilepsy in the foreseeable future.

## Author Contributions

GC: contributed to the conception and drafting the manuscript, ZZ: drafting the manuscript. MW and YG: revising the manuscript. BJ: contributed to the conception, drafting the manuscript, and final approval of the version to be published. TA: drafting the manuscript and revising the manuscript. All authors contributed to the article and approved the submitted version.

## Conflict of Interest

The authors declare that the research was conducted in the absence of any commercial or financial relationships that could be construed as a potential conflict of interest.

## Publisher's Note

All claims expressed in this article are solely those of the authors and do not necessarily represent those of their affiliated organizations, or those of the publisher, the editors and the reviewers. Any product that may be evaluated in this article, or claim that may be made by its manufacturer, is not guaranteed or endorsed by the publisher.
